# Molecular and Cellular Dynamics in the Skin, the Lymph Nodes, and the Blood of the Immune Response to Intradermal Injection of Modified Vaccinia Ankara Vaccine

**DOI:** 10.3389/fimmu.2018.00870

**Published:** 2018-04-25

**Authors:** Pierre Rosenbaum, Nicolas Tchitchek, Candie Joly, Lev Stimmer, Hakim Hocini, Nathalie Dereuddre-Bosquet, Anne-Sophie Beignon, Catherine Chapon, Yves Levy, Roger Le Grand, Frédéric Martinon

**Affiliations:** ^1^Immunology of Viral Infections and Autoimmune Diseases, IDMIT Department, CEA – Université Paris Sud 11 – INSERM U1184, Fontenay-aux-Roses, France; ^2^Vaccine Research Institute, Henri Mondor Hospital, Créteil, France; ^3^CEA – INSERM, MIRCen, UMS27, Fontenay-aux-Roses, France; ^4^INSERM U1169, Kremlin-Bicêtre, France; ^5^INSERM U955, Henri Mondor Hospital, University of Paris East, Créteil, France

**Keywords:** vaccine, modified virus Ankara, inflammation, systems biology, non-human primate, skin, lymph node, blood

## Abstract

New vaccine design approaches would be greatly facilitated by a better understanding of the early systemic changes, and those that occur at the site of injection, responsible for the installation of a durable and oriented protective response. We performed a detailed characterization of very early infection and host response events following the intradermal administration of the modified vaccinia virus Ankara as a live attenuated vaccine model in non-human primates. Integrated analysis of the data obtained from *in vivo* imaging, histology, flow cytometry, multiplex cytokine, and transcriptomic analysis using tools derived from systems biology, such as co-expression networks, showed a strong early local and systemic inflammatory response that peaked at 24 h, which was then progressively replaced by an adaptive response during the installation of the host response to the vaccine. Granulocytes, macrophages, and monocytoid cells were massively recruited during the local innate response in association with local productions of GM-CSF, IL-1β, MIP1α, MIP1β, and TNFα. We also observed a rapid and transient granulocyte recruitment and the release of IL-6 and IL-1RA, followed by a persistent phase involving inflammatory monocytes. This systemic inflammation was confirmed by molecular signatures, such as upregulations of IL-6 and TNF pathways and acute phase response signaling. Such comprehensive approaches improve our understanding of the spatiotemporal orchestration of vaccine-elicited immune response, in a live-attenuated vaccine model, and thus contribute to rational vaccine development.

## Introduction

Vaccine innovation would be aided by a better understanding of the basic mechanisms associated with processes that affect the duration, breadth, and efficiency of the host response, particularly the very early events that can be manipulated by antigen targeting, adjuvants and immune stimulants, dose, and delivery. Focusing on very early steps of the host response may also aid in finding ways to reduce reactogenicity to injected-material, which may limit vaccination acceptance, particularly for young children. Vaccination strategies have been improved by taking advantage of recent knowledge on pathogen sensing by key cells, such as dendritic cells (DCs), through pattern recognition receptors (PRRs) (1, 2). We and others have demonstrated that modifying the targeting of vaccine antigens to various DC receptors or blocking key cytokines at the injection site significantly affects the induced adaptive immune response (3–6). The interplay between innate and adaptive immunity is characterized by many other highly dynamic molecular and cellular modifications. Emerging ‘omic technologies may help to increase our knowledge and aid in the development of new immunization strategies (7, 8). The innate response in the blood can be triggered as early as 24 h after immunization when using adjuvants such as alum or toll-like receptor (TLR) (9) or after immunization with Modified virus Ankara (MVA) (10–13). These works suggest that very early events can have a strong impact to long-term responses to vaccines.

Skin is an ideal target for vaccine injection due to the diversity of resident and recruited immune cells, including macrophages, Langerhans cells, and several subsets of dermal DCs (14, 15). These cells, which express a wide variety of PRRs (16), can be used to tune the immune profiles of vaccines (17). Nevertheless, despite many advantages, intradermal (i.d.) route remains rarely used in the medical field due to the lack of reliability of the immunization technique (18). It remains, however, widely used in anti-tuberculosis vaccination to minimize the toxicity of the Bacille Calmette et Guérin (19), and for rabies vaccines (20). In addition, i.d. route may contribute to dose sparing (21–23), due to the efficiency of local antigen-presenting cells (APCs) (6, 10), which could provide a decisive advantage for products with scale-up production constraints. Delivery technologies are developed in order to enable skin vaccination (24).

Here, we characterized the early responses induced by i.d. injection of the MVA (25) attenuated vaccine into non-human primates (NHPs). This attenuated vaccinia virus has been safely used as a Smallpox vaccine in humans (26) and is a promising vector for recombinant vaccines (27–30). This vaccine has been found to be well tolerated and highly immunogenic, making it a good model of attenuated vaccine to describe immune responses. The use of NHPs was particularly relevant because the organization of their immune system is highly similar to that of humans and their response following vaccination is highly predictive of the response in humans (31–33).

In this study, we aimed to better understand the impact of early innate parameters on the elaboration of the adaptive response in a live attenuated vaccine model administered in the skin. The first step of the study consisted in an in-depth characterization of the innate immune responses in key localizations, i.e., injection site, lymph node (LN), and blood. The second step was to integrate data by using tools derived from systems vaccinology to identify key factors and to improve the knowledge of the dynamic of the interactions between immune parameters.

## Materials and Methods

### Ethics Statement

Adult male cynomolgus macaques (*Macaca fascicularis*), imported from Mauritius, weighing from 4 to 6 kg, were housed in the CEA animal facilities (Fontenay-aux-Roses, France). The macaques were handled in accordance with national regulations (Permit Number A 92-32-02), in compliance with the Standards of the Office for Laboratory Animal Welfare (OLAW) under Assurance number A5826-01, and the European Directive (2010/63, recommendation No. 9). This project received the government authorization Number 12-013. Interventions were performed by veterinarians and staff of the “Animal Science and Welfare” core facility after sedation with ketamine hydrochloride (10 mg/kg, Imalgen^®^, Rhône-Mérieux, Lyon, France).

### Intradermal Immunization

Prior to injection, the skin was shaved or depilated and cleaned with ethanol 70%. Each animal simultaneously received a total of 10 i.d. injections containing 4 × 10^7^ PFU each of a rMVA expressing eGFP (MVATG15938, Transgene SA, Illkirch-Graffenstaden, France) in 200 µl, equally divided between two areas: (i) 5 to 10 cm from the left inguinal LN and (ii) on the top left of the back.

### Tissue and Blood Samples

Prior to biopsies, the skin and inguinal area were cleaned with povidone iodate solution (Vetedine^®^, Vetoquinol SA, Lure, France). Biopsies of 8 mm in diameter were collected at untreated sites and 24, 48, and 72 h after injection. A total of eight skin biopsies were performed for each animal (Figure S1 in Supplementary Material). These biopsies induced insignificant inflammatory responses in comparison to i.d. injection of MVA (Figure S2 in Supplementary Material). The inguinal LNs were collected 24 h post injection. Blood was collected in K3-EDTA tubes (Greiner Bio-One, Frickenhausen, Germany) for cell blood counting (HmX, Beckman Coulter, Roissy, France), plasma collection, and flow cytometry. Plasma for cytokine analysis was collected after 10 min of centrifugation at 2,000 *g*. Blood (500 µl) collected in lithium heparin (Vacutainer BD) was mixed with 1 ml tempus RNA (Thermofischer Scientific, Waltham, MA, USA) for transcriptomic analysis. Tissue biopsies for transcriptomic profiling were preserved in 1 ml RNA later solution (Qiagen, Hilden, Germany) at 4°C overnight and then stored at −20°C.

### Extraction of Cells From Tissue Biopsies

Skin and LN biopsies were washed with PBS, weighed, cut into small pieces, and digested at 37°C under agitation (80 rpm) for 60 and 15 min, respectively, in 2 ml DMEM (Thermofischer Scientific) supplemented with 5% FCS (Lonza, Basel, Switzerland), 1% Penicillin/Streptomycin/Neomycin (Thermofischer Scientific), 10 mM Hepes (Thermofischer Scientific), 2 mg/ml collagenase D (Roche, Basel, Switzerland), and 0.02 mg/ml DNAse I (Roche). Tissue was then filtered using a 70-µm cell strainer and centrifuged. Supernatants were collected for cytokine analysis. Leftover tissue was shredded using a GentleMACs^®^ dissociator (Miltenyi Biotec, Paris, France). The cell suspension was then washed with PBS and stained for flow cytometry analysis.

### Flow Cytometry

All steps of the staining were performed at 4°C. Cell suspensions were first stained with viability dye LiveDead^®^ (Thermofischer Scientific) for 15 min, then washed with PBS. Fc receptors were blocked using PBS supplemented with 5% macaque serum for 20 min. Cell suspensions were stained for 30 min with 90 µl antibody mix (Table 1) diluted in BD Horizon Brilliant^®^ Stain Buffer (BD Biosciences, Franklin Lakes, NJ, USA). Cells were then washed twice in PBS and fixed using 150 µl BD Cellfix^®^ (BD). Red blood cells were removed with 1 ml BD FACs Lysing^®^ (BD) for 10 min at room temperature and washed twice with PBS. The data were normalized to the weight of the initial biopsy for the analysis of cell suspensions from tissues. Data from blood cells were normalized to the complete blood counts. The design of the cytometry panel and the rationale for gating strategies (Figure S3 in Supplementary Material) has been previously described (34).

**Table 1:** Panels of antibodies used for flow cytometry staining.

Specificity	Clone	Flurochrome of the Ab in the mix for		
		Skin cells	LN cells	Blood
CD1a	O10	AF700	AF700	N/A
HLA-DR	G46-6	APC-Cy7	APC-Cy7	V500
CD163	GHI/61	BV711	BV711	N/A
CD11c	3.9	BV510	BV510	PE-Cy7
CD45	DO58-1283	PerCp	N/A	N/A
CD123	7G3	N/A	PercP	PercP
CD66abce	TET2	APC	APC	FITC
CD3	SP34-2	V450	V450	APC-Cy7
CD8	RPA-T8	N/A	N/A	BV650
CD20	2H7	V450	V450	APC-Cy7
CD11b	Bear 1	PE-Cy7	PE-Cy7	A700
CD16	3G8	ECD	ECD	APC
NKG2a	Z199	PE	PE	PE
CD14	M5E2	N/A	N/A	V450
CD33	AC104.3E3	N/A	N/A	PE

### Confocal Time-Lapse Microscopy of rMVA Infection in the Skin

Ten micrograms of anti-HLA-DR AF700 (Biolegend) diluted in PBS were injected i.d., at the same time as the rMVA-eGFP injection, 4 h prior to biopsy at each injection site destined for confocal video-microscopy. Following fat tissue removal, skin biopsies were imaged for 18 h as previously published (35). The number of GFP^+^ cells and HLA-DR^+^ cells was calculated using Volocity^®^ software (PerkinElmer, Coventry, England) after applying a fine filter and then excluding objects below 200 µm^3^ and those above 4,000 µm^3^.

### *In Vivo* Fibered Confocal Fluorescence Microscopy (FCFM) for rMVA Visualization in the Skin

*In vivo* FCFM was performed with a fibered confocal fluorescence microscope (Cellvizio^®^, Mauna Kea Technologies^®^, Paris, France). A probe that records the green fluorescence signal at 488 nm by sweeping the site of interest was applied two times during 30 s (36). GFP^+^ objects were counted in each frame (size > 20 μm^2^) using ImageJ^®^ software (37). The analysis was performed on the 50 frames containing the highest number of GFP^+^ objects for each observed site. For each time-point, the number of fluorescent cells of up to three injection sites was measured and is expressed as the mean ± SD of GFP^+^ cells.

### Histopathology

Skin and LN samples were fixed at 4°C in 4% paraformaldehyde for 24 h. Samples were stored in PBS at 4°C. Paraffin inclusion was performed using a Microm HMS740 (ThermoFischer Scientific) automated system by successively replacing the PBS with alcohol, xylene, and paraffin. The tissue was cut afterward using a microtome and deposited on slides. Hemalun Eosin coloration was performed using a Microm HMS740, alternating between xylene, alcohol, Hematoxylin (Labonord, Templemars, France), and Eosin (VWR international, Fontenay-sous-Bois, France) baths.

### Immunohistochemistry

Prior to staining, slides with 3 µm slices were incubated at 37°C overnight. Paraffin was removed by washing the slides with increasing concentrations of xylene (VWR), then ethanol (VWR), and water using a Microm HMS740. Unmasking was performed using citrate tempus (Diagnostic Biosystems, Pleasanton, CA, USA) in a 95°C bath for 20 min. The slides were then subjected to peroxidase blocking using 3% dihydrogenated water for 10 min (Sigma Aldrich, Saint-Louis, MO, USA) and saturated using 10% normal horse serum (Vector laboratories, Burlingame, CA, USA) and 5% BSA (Sigma Aldrich) in PBS for 30 min. Primary staining with anti-GFP antibody (7.5 µg per slide) in saturation solution (Abcam, Cambridge, UK) was then performed for 90 min followed by secondary staining with biotinylated anti chicken IgG antibody (Abcam) 1/200 in PBS for 30 min. ABC Complex using the Vectastain ABC Kit standard Elite (Vector) and diaminobenzidin (Vector) were applied to the slides following supplier recommendations for 30 and 5 min, respectively. Slides were then colored using a Microm HMS740 to successively transfer the slides to baths containing hematoxylin, water, and increasing concentrations of ethanol and xylene. The slides were washed three times for 5 min in a bath containing PBS between each step of the procedure.

### Local and Systemic Cytokine Releasing Analysis

Supernatants of the digestion medium of LN, skin, and plasma were stored at −80°C. Cytokine levels were measured using a 23-plex MAP NHP Immunoassay kit PCYTMG-40K-PX23^®^ (Millipore, Billerica, MA, USA) according to supplier instructions.

### RNA Isolation and Microarray Profiles

Biopsies were immediately immersed in 1/100 RLT-beta-mercaptoethanol lysis buffer (Qiagen, Courtaboeuf Cedex, France) and then disrupted and homogenized using a TissueLyser LT (Qiagen) and the RNA purified using a Qiagen RNeasy Micro Kit. Whole blood RNA was recovered in tempus tubes (ThermoFisher scientific) and RNA purified using the *Tempus*™ Spin RNA Isolation Kit (ThermoFisher scientific). For both purifications, contaminating DNA was removed using the RNA Cleanup step of the RNeasy Micro Kit. Purified RNA was quantified using an ND-8000 spectrophotometer (NanoDrop Technologies, Fisher Scientific, Illkirch, France) and RNA integrity evaluated on a 2100 BioAnalyzer (Agilent Technologies, Massy Cedex, France). cDNA was synthesized and labeled with biotin using Ambion Illumina TotalPrep RNA Amplification Kits (Applied Biosystem/Ambion, Saint-Aubin, France). Labeled cRNA were hybridized to Illumina Human HT-12V4 BeadChips. All steps were performed following the supplier instructions. Raw and normalized microarray data have been deposited in the ArrayExpress database (38) under an accession number E-MTAB-5907.

### Statistical Analysis and Graphical Representations

Cytometry, Luminex, and transcriptomic data were analyzed using R (39). Hierarchical clustering was performed based on the Euclidean metric using the Ward linkage method. Flow cytometry and Luminex measurements were normalized for heatmap representations. Differentially expressed genes were identified using the paired Student’s *t*-test (*q*-value < 0.01), and based on a 1.2-fold change threshold to discard genes having very low amplitude of down- or upregulations. Multidimensional scaling (MDS) representations were generated using the singular value decomposition (SVD)-MDS algorithm (40). Kruskal Stress (41) quantifies the quality of the MDS as the fraction of information lost during the dimensionality reduction process. Functional enrichment analysis of the differentially expressed genes was performed using Ingenuity Pathway Analysis software (IPA^®^, Qiagen, http://www.qiagen.com/ingenuity). Statistical analysis for flow cytometry and Luminex data were performed using Prism^®^ 6.0 (Graphpad Software Inc., La Jolla, CA, USA). Two-sided Friedman tests followed by a Dunn’s posttest comparing each time-point with the baseline were performed. Significant correlations between Luminex, transcriptomic, and cytometry variables were identified using the Pearson correlation coefficient based on a correlation threshold of 0.80 and a *q*-value threshold of 0.05. Representation of the co-expression network was generated using Cytoscape^®^ (42).

## Results

### Skin Infection With MVA Attenuated Vaccine Induces Localized Inflammatory Immune Response

**Figure 1:**
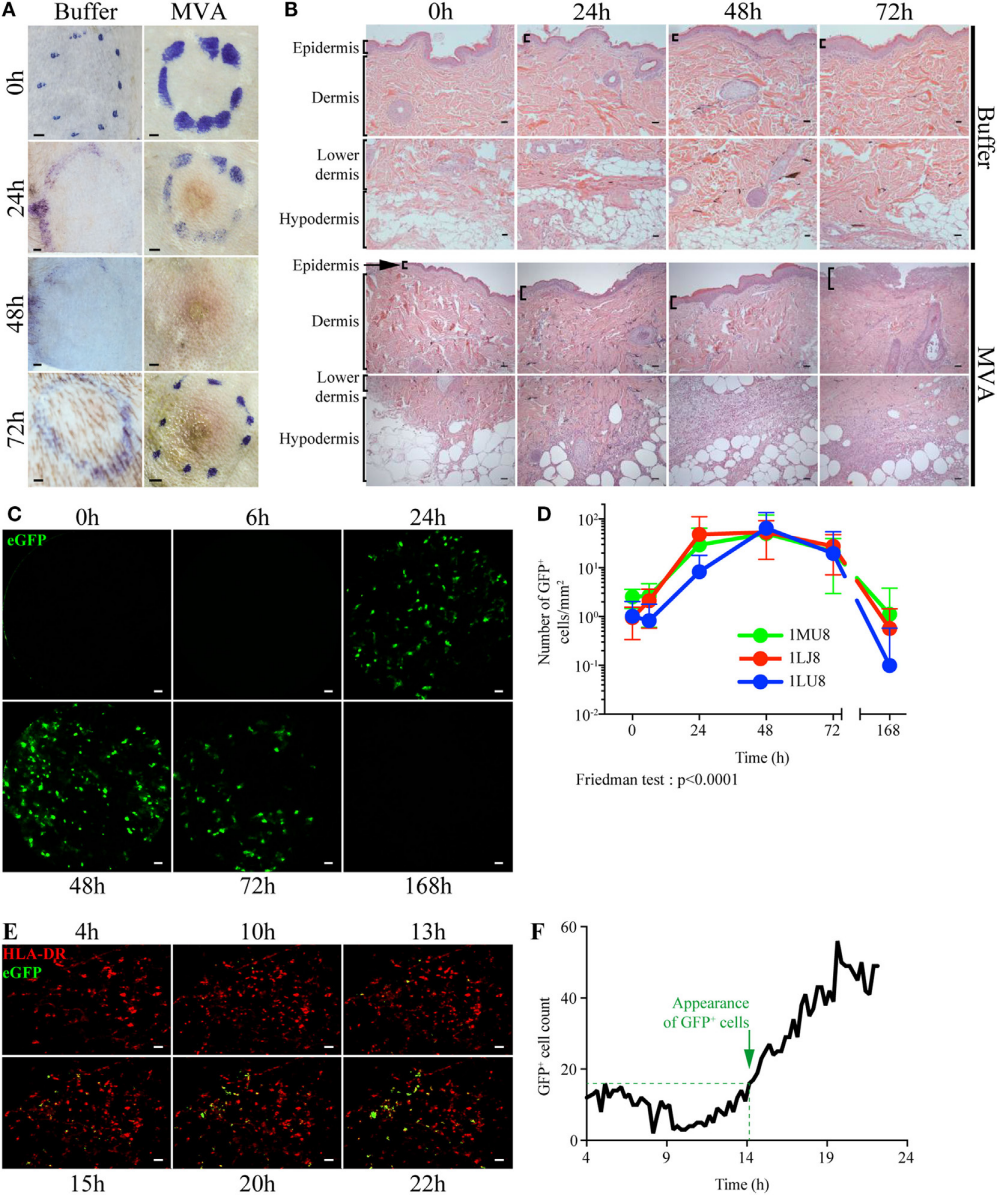
Dynamics of local inflammation and infection after intradermal injection of rMVA. **(A)** Image of the skin before and after Buffer or rMVA injection. Scale bars correspond to 2 mm. **(B)** HE staining of transversal sections of non-human primate skin biopsies taken at the site of injection before or 24, 48, or 72 h after Buffer or rMVA injection. Scale bars correspond to 100 µm. **(C)** Representative images recorded by *in vivo* confocal endo-microscopy. Recordings were performed at the site of injection in the dermis (at a depth of 100 ± 35 µm in the skin) with a probe detecting fluorescent signals after excitation at a wavelength of 488 nm. Green fluorescence corresponds to the production of GFP in rMVA-infected cells. Scale bars correspond to 50 µm. **(D)** Kinetic profile of GFP^+^ cells in the skin after rMVA injection. The graph represents the mean ± SD of the number of GFP^+^ cells/mm^2^ in the 50 frames with the most GFP^+^ cells. **(E)** Representative images from time-lapse video-confocal microscopy of HLADR^+^ (in red) and GFP^+^ (in green) cells after i.d. injection of rMVA. Skin biopsies were observed for 22 h p.i. Acquisition was performed at a depth of 50–150 µm in the skin (dermis) corresponding to a stack of 10 images. Visual representations correspond to a two-dimensional visualization of this stack at the indicated time p.i., after cropping the region of interest. Scale bars correspond to 20 µm. **(F)** Kinetic profile of GFP^+^ cell detection in the dermis during the first 22 h p.i. The graph indicates the number of GFP^+^ cells counted for each image (1 image each 15 min). Green objects between 200 and 4,000 µm^3^ were considered to be GFP^+^ cells. The approximate time of appearance of non-background GFP^+^ cells is indicated with the dotted line at 14–15 h p.i.

**Figure 2:**
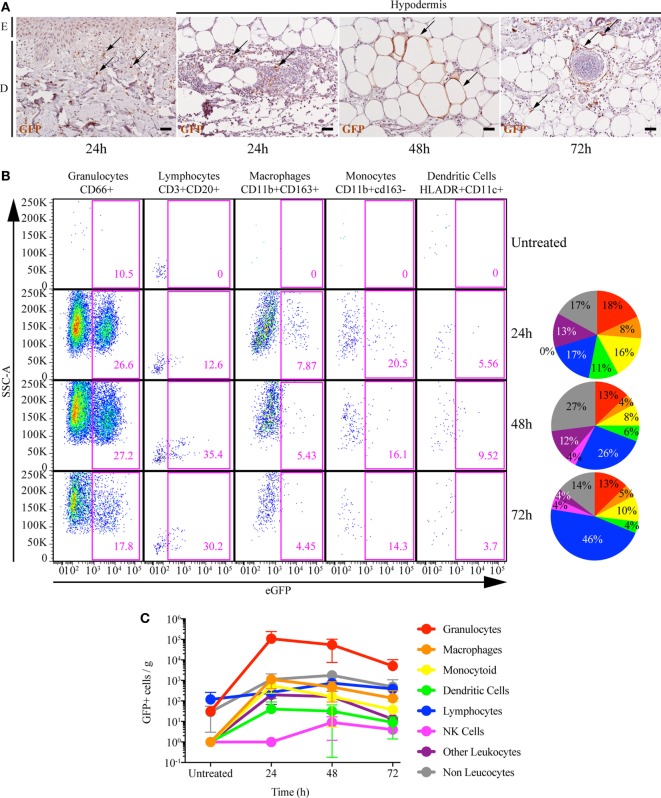
rMVA infects a wide range of skin cell types. **(A)** *In situ* localization of cells infected with rMVA. Transversal sections of paraffin-embedded skin biopsies were stained with an anti-GFP antibody and then colored with HE. E and D indicate the epidermis and the dermis, respectively. Scale bars correspond to 100 µm. Arrows indicate GFP^+^ cells as example. **(B)** Percentage of rMVA-infected cells in the skin. The dot plots show the evolution of recruitment of the main immune cells to the skin and the percentage of GFP^+^ cells. **(C)** Number of GFP^+^ cells per gram of skin biopsy. The numbers of GFP^+^ cells in the indicated cell population were normalized to the weight of the skin biopsies. The graph represents the mean ± SD (*n* = 3). See also Figure S3 in Supplementary Material for gating strategies.

Mantoux i.d. injection with rMVA expressing eGFP induced mild local lesions, detected as early as 24 h post injection (p.i.), which progressively evolved into an erythema (Figure 1A). Epidermal thickness increased by 24 h p.i. followed by mild parakeratosis 48 h p.i. and epidermal cell necrosis at 72 h p.i. (Figure 1B). In the dermis, we observed edema, associated with moderate perivascular neutrophilic and macrophage infiltration from 24 to 72 h p.i. There was a massive neutrophil and macrophage infiltration at the interface between the lower dermis and the hypodermis, associated with necrosis. The eGFP-expressing cells resulting from rMVA infection were tracked in the skin by repeated *in vivo* imaging using FCFM (Figure 1C). Expression of eGFP was detected as early as 24 h p.i., peaked at 48 h (56.2 ± 59.3 cells per mm^2^), persisted at a high level until day 3 (22.8 ± 24.7 cells per mm^2^), and disappeared by day 7 (Figure 1D). This kinetic profile was confirmed by confocal time-lapse microscopy (Figure 1E). In skin biopsies collected 4 h after *in vivo* injection of the rMVA, the first eGFP signals were detected 13–15 h p.i. and reached the maximum level by 18–22 h (Figure 1F). Some, but not all, eGFP^+^ cells also expressed HLA-DR indicating that the detection of the recombinant gene was not limited to skin APCs. *In situ* analysis revealed the heterogeneous morphology of the eGFP^+^ cells, which included DCs, macrophages, adipocytes, and granulocytes (Figure 2A). Most were localized to vascularized areas, such as the papillary–reticular dermis interface and the hypodermis layer. No eGFP^+^ cells were detected in the epidermis. We further characterized the phenotype of the eGFP^+^ skin cells by performing flow cytometry on cell suspensions obtained from the injection site, including the epidermis and dermis. Cells containing locally produced eGFP reached a peak between 24 (25.8 ± 4.9% of total living cells) and 48 h (19.5 ± 3.1%) p.i., persisted through 72 h (6.4 ± 5.9%), and completely disappeared by 168 h (Figure S4 in Supplementary Material), confirming our previous observations. The rMVA exhibited considerable cellular pleiotropism (Figure 2B) *in vivo* in NHPs, confirming previously reported observations (43, 44). The major targeted populations were granulocytes, monocyte/macrophages, and lymphocytes with the proportion of infected cells ranging from 3 to more than 30% (Figure 2B). Granulocytes represented most of the locally recruited cells and were by far the predominant population containing eGFP antigen when normalized to the number of cells per gram of tissue (Figure 2C). It is thus likely that this population significantly influences the mechanisms that affect installation of the vaccine-specific response. The proportion and number of eGFP^+^ DCs, which are major resident sentinels in the skin, was low.

### rMVA Injection Induces Strong Local Production of Innate and Adaptive Mediators

**Figure 3:**
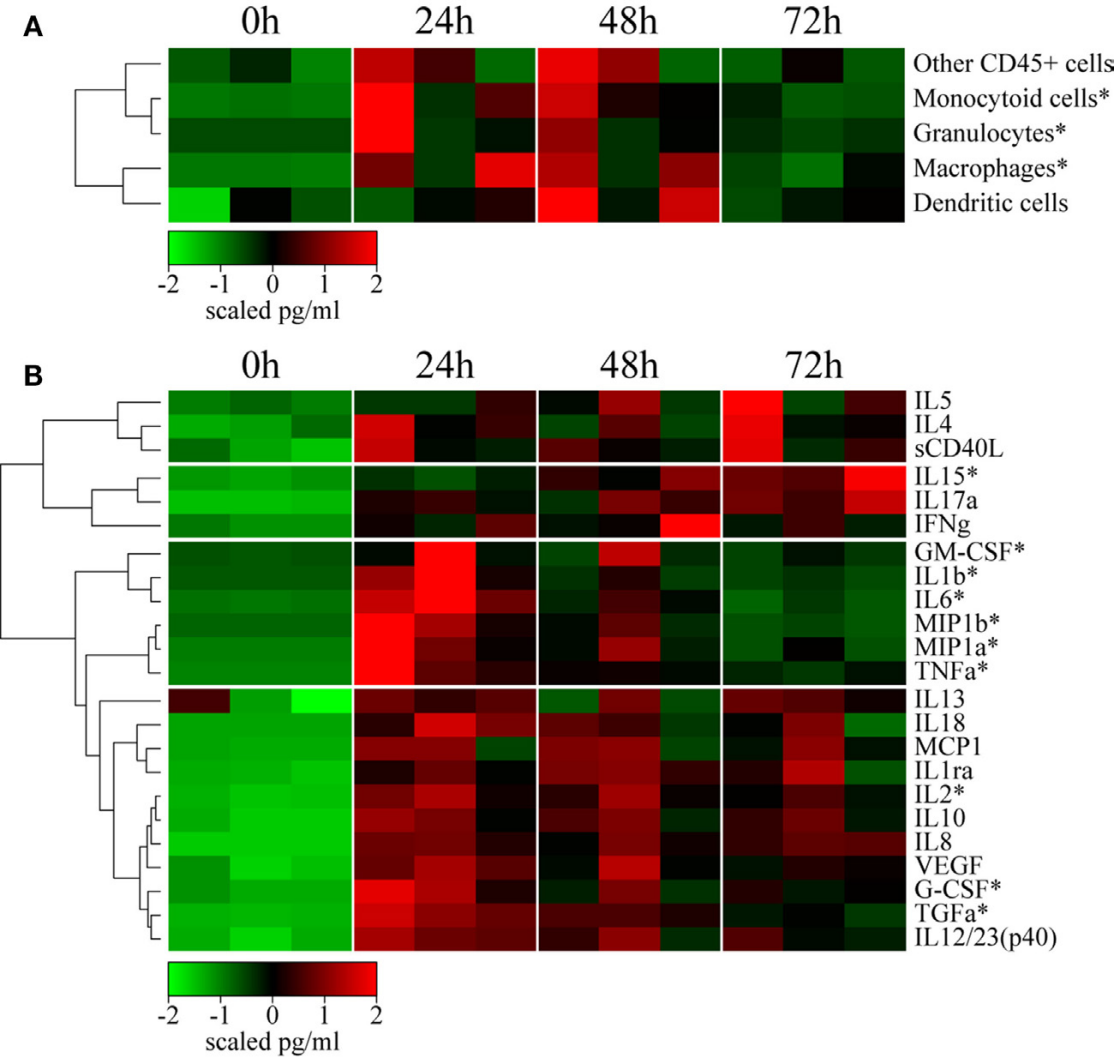
rMVA injection induces local cellular trafficking and cytokine release. **(A)** Heatmap representation of the cell counts in skin subset populations discriminated by flow cytometry. Cell populations were automatically sorted following hierarchical clustering represented by the dendrogram on the left. The number of cells was calculated by dividing the number of events by the weight of the biopsy. Values were standardized to display the same range of expression values for each cell population to properly visualize the cell population kinetics. See Figure S3A in Supplementary Material for gating strategies. **(B)** Heatmap representation of cytokine release in the skin after rMVA injection. Cytokine expression was automatically sorted following hierarchical clustering represented by the dendrogram. Values were standardized to display the same range of expression values for each cytokine to properly visualize the cytokine production kinetics. *0.05 > *p*-value > 0.01 in Friedman’s test over time.

Local cellular recruitment and cytokine production were found to very similar over the kinetics between the three animals. Cells recruited to the site of injection included mainly inflammatory myeloid cells, with statistically significant increases in the number of macrophages, granulocytes, and monocytoid cells (Friedman test; *p* < 0.05), which increased at 24 and 48 h p.i. compared to baseline (Figure 3A). As expected, the recruitment of inflammatory cells was associated with the local release of inflammatory cytokines and chemokines (Figure 3B). Local levels of a first cluster of pro-inflammatory molecules, including GM-CSF, IL-1β, IL-6, MIP1α, MIP1β, and TNFα, significantly increased (*p* < 0.05) as early as 24 h p.i. (no earlier time-points were tested), but then rapidly decreased to baseline levels by 72 h. A second cluster of inflammatory cytokines, including MCP1, IL-12/23(p40), IL-8, IL-18, and G-CSF, remained high through 72 h (no later time-point was tested), in contrast to the previous group. These molecules certainly reflect macrophage and granulocyte activity. We also observed an increase in the level of the angiogenic factors TGFα and VEGF, also part of the inflammation process. This cluster also included anti-inflammatory mediators, such as IL-10 and IL-1RA, for which prolonged expression is expected. Finally, cytokines related to an adaptive response, including IL-2 and IL-13, were also detected in this cluster as early as 24 h p.i., indicating that local cells may contribute to the installation of the adaptive response, although no T or B cell recruitment was observed. Adaptive response mediators, including IFN-γ, IL-5, IL-4, IL-15, and IL-17α, were also present in a third cluster, detectable as early as 24 h p.i., which appeared to steadily increase until 72 h p.i. This group also included soluble CD40L, indicating that immunosuppressive factors are also induced, probably as a mechanism to limit the local reaction.

### Impact of rMVA Intradermal Injection on Draining LNs

**Figure 4:**
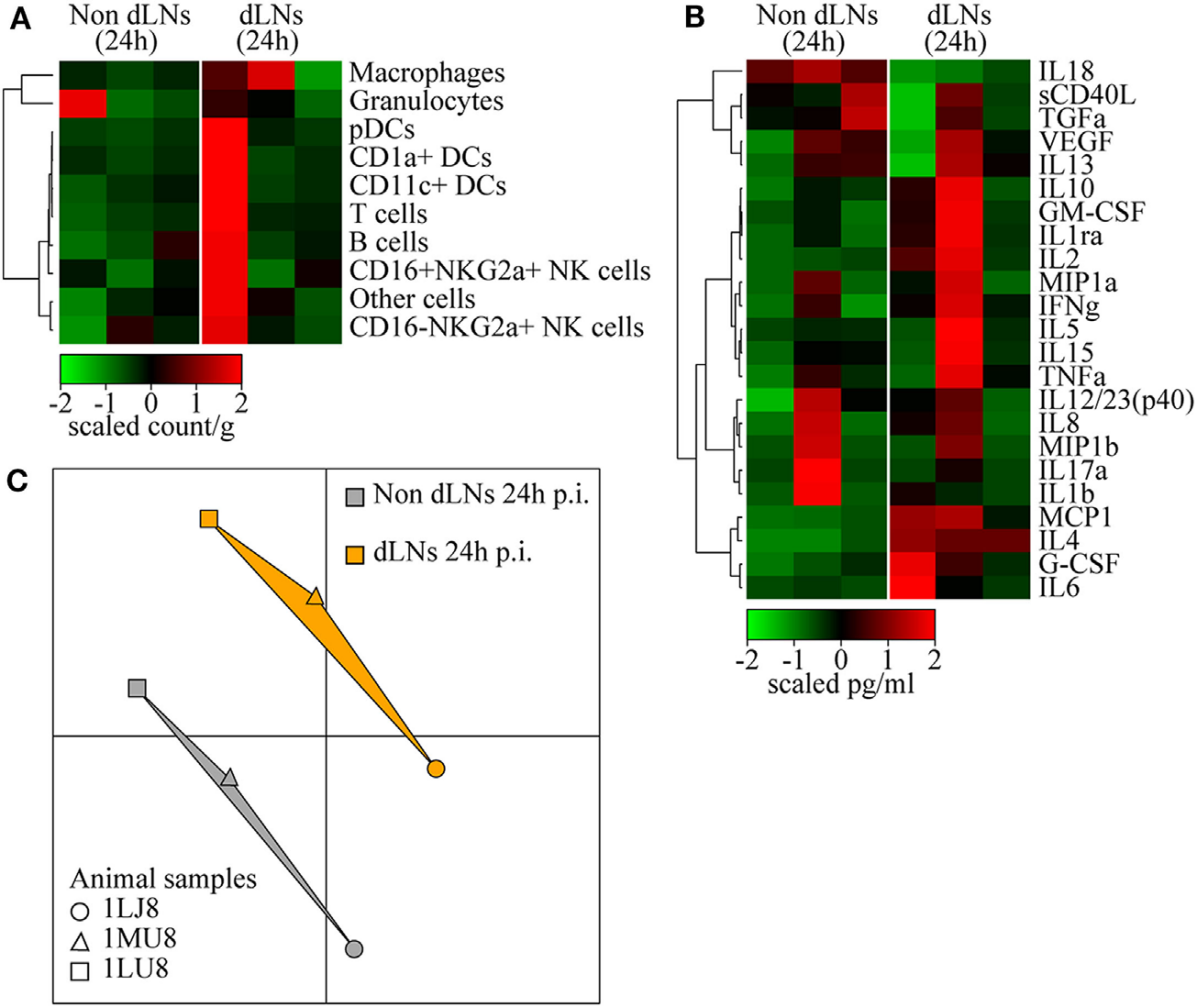
Immune reaction in the draining lymph nodes (LNs). **(A)** Heatmap representation of cell populations discriminated by flow cytometry in the inguinal LNs draining the sites of rMVA injection (dLNs) and the contralateral LNs (Non dLNs). Cell populations were automatically sorted following hierarchical clustering represented by the dendrogram on the left. The cell count/gram was calculated by dividing the number of events by the weight of the biopsies, which were collected 24 h p.i. Values were standardized to display the same range of expression values for each cell population to properly visualize the cell population kinetics. See Figure S3B in Supplementary Material for gating strategies. **(B)** Heatmap representation of cytokine release in the LNs after rMVA injection. Cytokine production was automatically sorted following hierarchical clustering represented by the dendrogram. Values were standardized to display the similar range of expression for each cytokine to properly visualize the cytokine production kinetics. **(C)** Multidimensional scaling representation of transcriptomic signatures in LNs 24 h after rMVA injection. Biological samples are represented as dots in a two-dimensional space. The distances between the dots are proportional to the Euclidian distances between the transcriptomic profiles.

We collected LNs draining the vaccine injection site 24 h p.i. to study the dynamics of the interaction between tissues in response to rMVA injection. The contralateral LNs were collected as a control. We did not observe significant differences between LNs, as only three animals per group were included at only one time-point. However, one of the animals appeared to have an increase in the proportion of macrophages and granulocytes in draining LNs (Figure 4A). This cell recruitment profile reflected the local increases observed in the skin. In addition, this animal also showed an increase in the proportion of APCs, such as CD1a+ DCs, as well as T lymphocytes (Figure 4A). Despite important heterogeneity, cytokine levels measured in total LN tissue extracts indicated increased levels of inflammatory cytokines, such as IL-6, TNFα, and particularly MCP1, G-CSF, and IL-8 (Figure 4B). This is consistent with the migration of macrophages and granulocytes. Additional cytokines, including IL-4, IFN-γ, IL-2, and IL-13, related to adaptive responses were highly elevated in the draining LNs. Inversely, IL-18 was found to be consistently decreased after MVA immunization. Finally, analysis of transcriptomic profiles showed increased expression of genes associated with the initiation of immune responses in the LN draining the vaccine injection site. In details, we found 49 genes differentially expressed between MVA and control samples, 15 of them were upregulated while 34 of them were downregulated. A pathway enrichment analysis was performed on this differentially expressed gene set. We identified one significantly enriched upstream transcriptional regulator, ATF3, associated with TLR4 signaling (45). Other markers of activation also appeared to be upregulated in the draining LN without reaching a significant threshold likely because of the limited sample size. We used MDS representations to better visualize similarities and dissimilarities between transcriptomic profiles. This approach provides superior fidelity in representing the distance between different instances when analyzing high-dimensional geometric objects than more classical SVD-based methods (40). MDS analysis clearly segregated the transcriptomic signature of the draining LNs from that of the contralateral LNs (Figure 4C).

### From Local Reactions to Systemic Vaccine Responses

**Figure 5:**
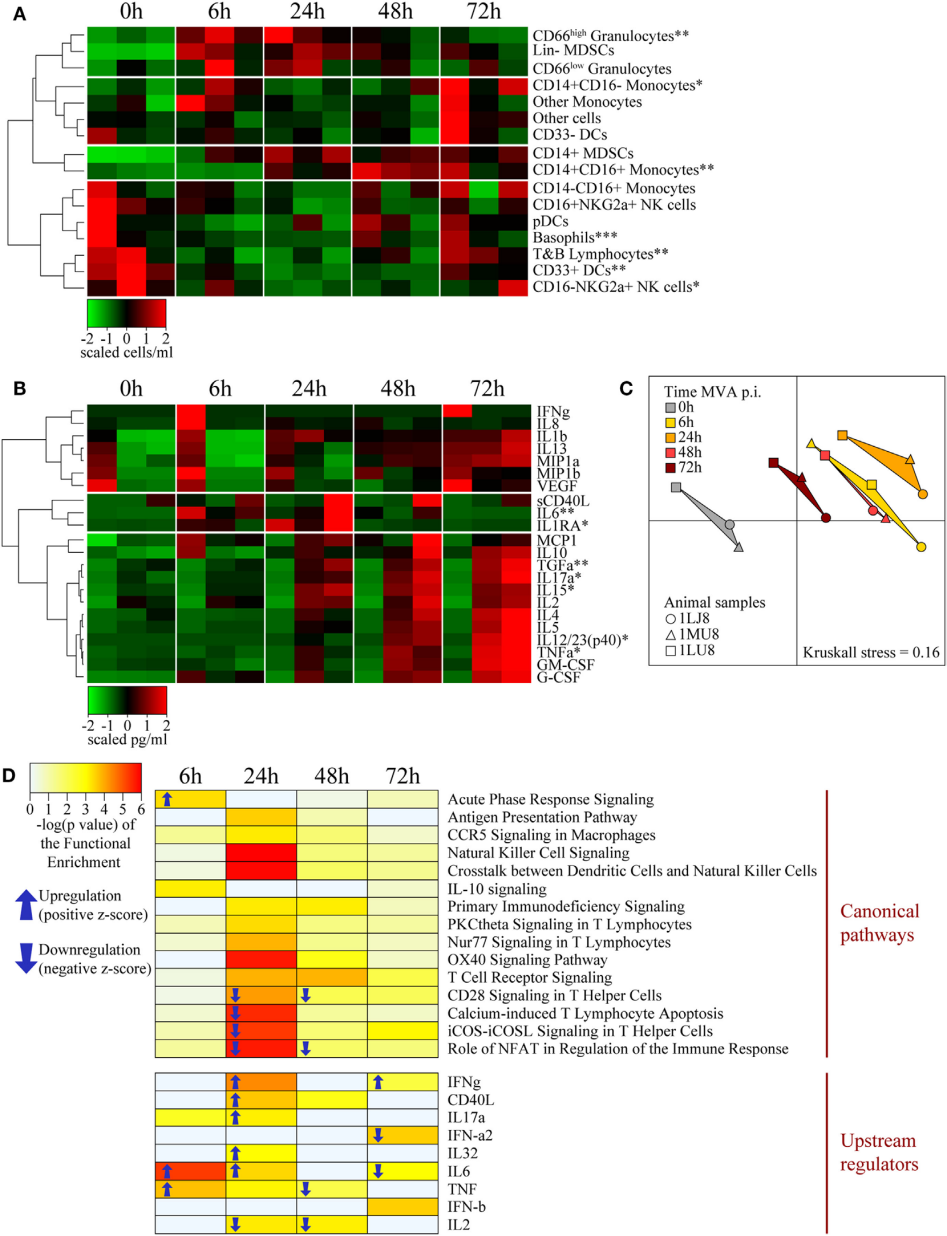
Systemic effects of intradermal rMVA injection. **(A)** Heatmap representation of blood cell populations discriminated by flow cytometry. Cell populations were automatically sorted following hierarchical clustering represented by the dendrogram on the left. The number of cells/milliliter was calculated using the number of leukocytes obtained by complete blood count analysis. Values were standardized to display the same range of expression values for each cell population to properly visualize the cell population kinetics. See Figures S3C,D in Supplementary Material for gating strategies. **(B)** Heatmap representation of cytokine release in the blood after rMVA injection. Plasma cytokine titrations were automatically sorted following hierarchical clustering represented by the dendrograms on the left. Values were standardized to display the same range of expression for each cytokine to properly visualize the cytokine production kinetics. **(C)** Multidimensional scaling representation of transcriptomic signatures in the blood after rMVA injection. Biological samples are represented as dots in a two-dimensional space. The distances between the dots are proportional to the Euclidian distances between the transcriptomic profiles. **(D)** Heatmap representation of functional enrichment. Data were extracted from differentially expressed genes relative to the baseline condition and functional enrichment expressed as −log(*p*-value), with a cutoff −log(*p*-value) > 3 for at least one time-point. Only differentially expressed canonical pathways and upstream regulators linked to the immune response are represented. Arrows correspond to the sign of the *z*-score, indicating the orientation of the expression of the group of genes (*n* = 3). *0.05 > *p*-value > 0.01; **0.01 > *p*-value > 0.001; ***0.001 > *p*-value in Friedman’s test over time.

We examined the effect of local cellular and molecular events on systemic immunity in the blood of vaccinated animals. The number of blood CD66^high^ granulocytes significantly increased (*p* < 0.05) very early (6 h) following vaccine injection (Figure 5A), consistent with the inflammation described in the skin and the draining LN. The level of these cells peaked from 6 to 24 h p.i. and then rapidly decreased to reach baseline levels by 72 h p.i. The increase in granulocyte levels in the blood preceded their recruitment to the skin. Classical monocyte (CD14^+^CD16^−^) numbers also increased in the blood by 6 h p.i., but then rapidly decreased by 24 h p.i., probably due to their differentiation into pro-inflammatory (CD14^+^CD16^+^) and non-classical (CD14^−^CD16^+^) monocytes, which indeed increased by 24 and 72 h, respectively. The increase in classical monocyte numbers also preceded the recruitment of monocyte/macrophages to the skin, similarly to granulocytes. In contrast to the myeloid populations, the number of T and B lymphocytes, NK cells, and pDCs strongly decreased in the blood early after vaccine injection (6 h), and then reappeared later, probably due to their migration to the sites of injection and draining lymphoid tissues. We also observed an increase (*p* = 0.0559) in the number of CD14^+^HLADR^−^CD33^+^ cells, expressing a phenotype corresponding to myeloid-derived suppressor cells (CD14^+^ MDSCs), at 24 h p.i., preceded by a transient increase in the number of early-stage Lin^−^ MDSCs-like cells (46), between 6 and 24 h p.i. Early appearance of these cells may represent a natural mechanism to control initial inflammatory processes.

Cytokine titration indicated that IL-6 levels transiently increase from 6 to 24 h in blood along with molecules associated with acute inflammation, such as IL-1β, MIP1α/β, and MCP1, which persisted in the blood through 72 h p.i. (Figure 5B). These cytokine profiles were similar to those observed in the skin. In contrast, TNFα clearly exhibited different kinetics in skin and blood, suggesting specific roles in the two compartments. We observed similar differences for IL-12 and TGFα. In addition, the levels of cytokines associated with adaptive responses, including IL-2, IL-4, IL-17α, IL-15, and IL-5, progressively increased up to the latest time-point (72 h) of the study. The cytokine titration profile in blood (Figure 5B) was clearly distinct from that of the skin (Figure 3B), suggesting that the cytokines found in the blood vessels of the skin had little, if any, influence on the cytokines measured in total tissue extracts.

We found a total of 742 genes differentially expressed in at least one time-point relative to control samples. A total of 123 genes were found to be down- or upregulated at 6 h, 354 genes at 24 h, 361 genes at 48 h, and 89 genes at 78 h. MDS analysis restricted to the set of differentially expressed genes in blood revealed a clear segregation between time-points (Figure 5C). The 24 h p.i. time-point was the most distant from the baseline. Gene expression levels 6 and 48 h p.i. showed an intermediate distance from baseline and had close gene expression profiles. Although the 72 h p.i. profile was the closest to the baseline values, it remained significantly distant. Functional enrichment analysis revealed that the expression levels of genes associated with several canonical pathways and upstream regulators were significantly modified (*p*-value < 10^−3^) in blood cells (Figure 5D). Genes associated with the inflammatory response (TNFα, IL-6, IL-32, and acute phase response signaling) were upregulated at early time-points, consistent with changes in the cellular composition in blood and cytokine levels in plasma. In contrast and consistent with T-cell migration out of blood circulation, canonical pathways and regulators that contribute to the lymphocytic response, such as IL-2, CD28 signaling, and iCOS-iCOSL signaling, were downregulated. Transcripts that regulate important innate canonical pathways, e.g., antigen presentation pathways, CCR5 signaling in macrophages, IL-10 signaling, or NK-DCs crosstalk, showed some change without any overall clear up- or downregulation (Figure 5D).

### Co-Expression Network Representation Highlights the Predominant Factors Involved in the Early Vaccine Response

**Figure 6:**
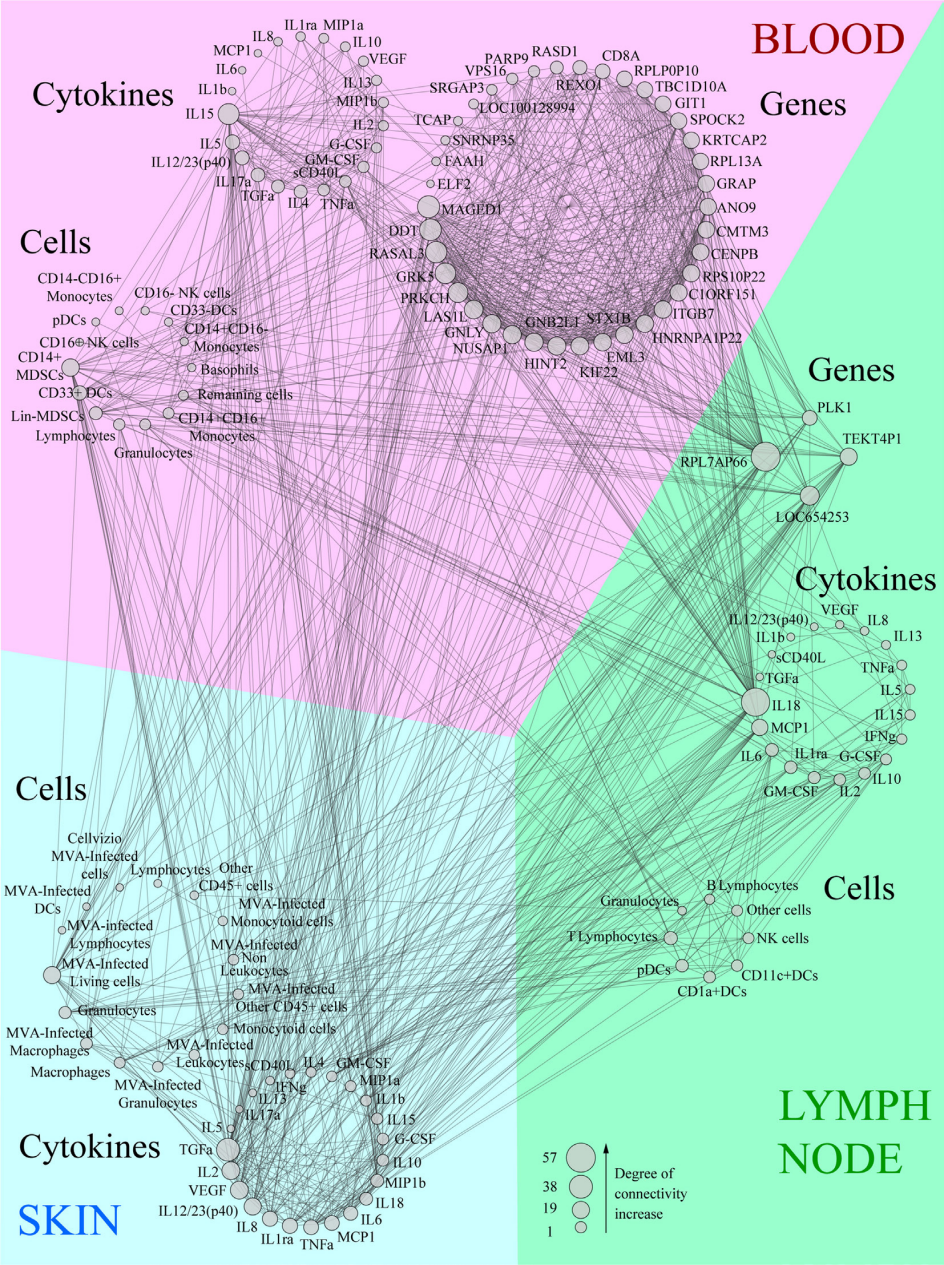
Co-expression network showing the correlations between each biological variable evaluated. Data were sorted in groups depending on the compartment (blood, lymph node, or skin) and type (cytokine, cell, or gene). Variables were then sorted depending on their size which is proportional to the number of correlations (degree of connectivity) they establish. All lines linking two parameters correspond to significant correlations between these variables (|*R*| > 0.8, *q*-value < 0.05).

**Figure 7:**
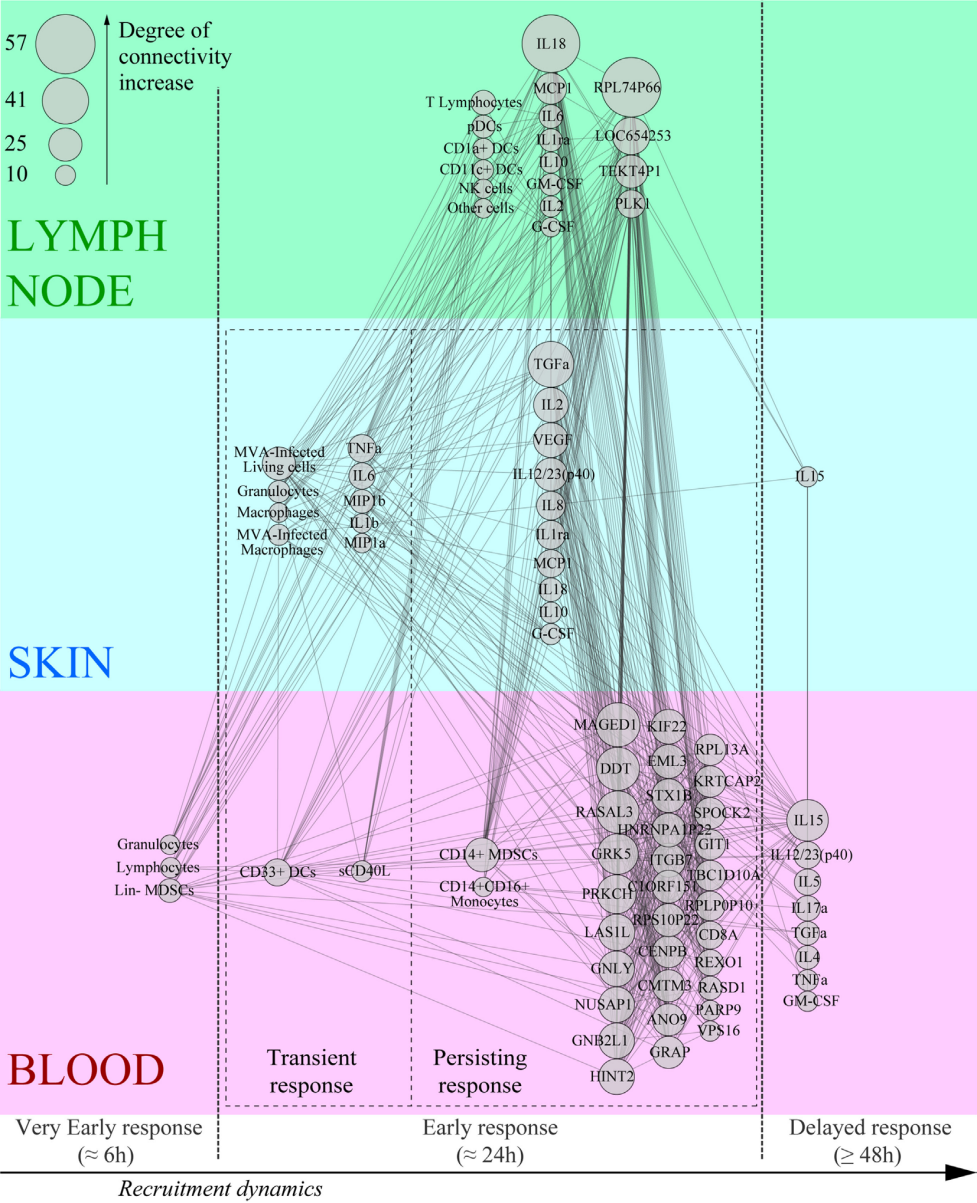
Co-expression network based on the kinetics of recruitment of key effectors of the Modified virus Ankara-induced innate response. The most highly correlated variables (degree of connectivity > 10) were sorted according to their compartment, their type, and their kinetics of expression after rMVA injection.

We integrated our data in a global analysis to provide a comprehensive overview of the early immune reaction following vaccination. We calculated Pearson correlations (cutoff: |*R*| > 0.8 and *q*-value < 0.05) for the whole dataset to establish links between the parameters of the three studied compartments (Tables S1 and S2 in Supplementary Material). This led to the construction of a co-expression network where the nodes correspond to the biological variables and the connecting lines correspond to significant correlations (degree of connectivity) between two variables (Figure 6). Non-draining LNs were considered to be the untreated conditions of draining LNs. The parameters were organized according to their type (cell, cytokine, or gene) and their origin (skin, blood, or LN). Parameters that did not lead to significant correlations are not represented. Cytokines measured in the skin strongly correlated with cytokines in draining LNs (48 connections), including inflammatory mediators such as TNFα, MIP1β, IL-6, and IL-1β, as well as factors associated with monocyte/macrophage and granulocyte recruitment, e.g., TGFα, G-CSF, and GM-CSF. Cytokines produced in the skin were strongly associated with gene products of the LNs (41 connections). However, three out of four of these gene products had unknown functions; the fourth (PLK1) is involved in cell proliferation. Blood and skin were also connected through their cytokines, but to a lesser extent (35 connections), with TNFα and TGFα emphasizing again the role of granulocytes and monocyte/macrophages and IL-17α, IL-15, IL-5, and IL-12/23 suggesting the impact of the injection site on installation of the adaptive response. In addition, CD14+ MDSCs from the blood strongly correlated with cytokines produced in the skin (11 connections). Gene products from the blood represented the largest group connected to the other parameters (38 genes with significant correlations). The gene products with the highest degree of connectivity were involved in the regulation of apoptosis (MAGED1), cell activation (DDT, GNLY, GNB2L1), cellular signaling pathways (RASAL3, PRKCH), cell mobility (GRK5), and cell proliferation (LAS1L, NUSAP1, HINT2, KIF22, EML3, CENPB, REXO1, PARP9, ELF2). In addition, these gene products from the blood were strongly connected to the pro-inflammatory cytokine IL-18 produced in the LNs (28 connections). We also organized the correlated biological parameters according to their peak of expression over the time following vaccine injection to evaluate the chronology of the events (Figure 7). This representation associated the blood, skin, and the LNs at 24 h p.i., at the peak of the inflammatory response, with monocyte/macrophages, granulocytes, and related cytokines as major actors. This kinetic profile confirmed the early involvement of actors of inflammation relative to those of the adaptive responses. In addition, it also showed that the control of the inflammatory responses was initiated early, with Lin^−^ MDSCs detected in the blood 6 h p.i. and CD14^+^ MDCS 24 h p.i.

## Discussion

Here, we aimed to better understand how cellular and molecular vaccine-induced innate immune parameters are interacting each other and how they could contribute elicit a vaccine response profile. To our knowledge, this study remains the first to characterize simultaneously local and systemic early kinetics in NHP model after vaccination. For this purpose, we used systems vaccinology approaches to integrate innate parameters for identifying key factors that can impact the adaptive responses. We used a multimodal analysis strategy to characterize the early steps of the immune response to MVA infection, at the local site of injection, in the draining LN, and blood. The MVA cell infection started 15 h p.i., involved several cell populations, and persisted for 3 days. The local innate response was characterized by early massive recruitment of granulocytes, macrophages, and monocytoid cells, associated with local production of GM-CSF, IL-1β, MIP1α, MIP1β, and TNFα. The innate response was also initiated at the systemic level with rapid and transient granulocyte recruitment and the release of IL-6 and IL-1RA, from 6 to 24 h p.i., followed by a persistent phase involving inflammatory monocytes. Systemic inflammation was confirmed by molecular signatures, such as those of genes that upregulate the IL-6 and TNF pathways and acute phase response signaling. Finally, integration of the data in a co-expression network allowed visualization of the relationships between the three compartments and the chronology of the events.

As a modified form of the vaccinia virus, MVA binds to a wide range of host cells and fuses with the cell membrane without the requirement of specific cell receptors, but the ability to fully complete the replication cycle is dependent on downstream intracellular events (44). The impaired ability of MVA to fully replicate in mammalian cells (47) has no apparent effect on recombinant gene expression (48). These properties are consistent with the detection of the recombinant gene (eGFP) in several cell subsets and the broad stimulation of innate immunity, suggesting that our observations are representative of diverse rMVA vectors expressing vaccine antigens, such as HCV, HIV, HBV, Ebola, malaria. By infecting a large variety of cells, MVA is widely sensed by several PRRs, such as TLR2, TLR6, MDA5, NALP3 inflammasome pathways (11), or cGAS (49). These PRRs are expressed in several resident cell types of the skin, including immune cells, as well as keratinocytes (50). Consequently, the recruitment of inflammatory cells, mostly originating from the blood, was expected as a part of the early response to the vaccine (6, 51).

We observed no clear-cut DC migration, which was possibly outshone by the magnitude of the inflammatory response. Pathogen sensing and the important cellular trafficking observed are likely associated through the microenvironment modifications mediated by local cytokine release, particularly G-CSF, GM-CSF, IL-1β, IL-6, MIP1β, and TNFα which contribute to the recruitment of granulocytes and macrophages and increase their cytotoxic activity and migration to the inflammation site. This localized cytokine release, along with local apoptosis of granulocytes, led to resolution of the inflammation (52), shown by the decrease of pro-inflammatory cytokine levels and the increase of IL-2 and IL-15, opening the way to potential local specific T-lymphocyte responses.

The local immune response to rMVA was associated with considerable systemic inflammation which was initiated before the detection of the first infected cells expressing the recombinant antigen (eGFP). We postulate that the first immune signals were delivered by resident skin cells through inflammatory eicosanoid mediators soon after rMVA administration (52), leading to rapid granulocyte release from the bone marrow and migration to the site of injection through the blood. The level of IL-6 and IL-1RA and the upregulation of acute response pathways 6–24 h p.i. may be associated with the increase of granulocytes in the blood. The downregulation of several pathways related to lymphocyte responses could be linked with a decrease of lymphocytes in the blood. In addition to lymphocytes, CD16^−^ NK cells and CD33^+^ DCs also left the systemic compartment, but were not observed at the site of injection. They may modify their phenotype upon migration and differentiation and later migrate directly to the LNs.

The correlations between the biological parameters suggest that the magnitude of the inflammatory response directly orchestrates the early steps of the adaptive response. This is illustrated by the early correlations between IL-2 levels in the skin and LNs. In addition to TNFα, this cytokine leads to a Th1 response, consistent with the primary T cell response profile induced by MVA (43, 53). We also identified IL-15 as an early key parameter detected in the skin and in the blood. This cytokine vastly associated with IL-2 and mainly produced by monocytes and macrophages could also modulate the immune response toward a Th1 profile (54). However, our analysis also revealed the coexistence of effectors of the Th2 response, such as IL-4, IL-5, IL-10, and IL-13, in the LN. Moreover, the level of the pro-inflammatory cytokine IL-18, which is associated with orientation toward the Th1 response, was decreased after MVA immunization and thus inversely correlated with several recruited cell types and cytokines. It may, however, be a signature of inflammasome activation in subcapsular macrophages before 24 h p.i and thus be consistent with MVA-induced responses observed in mouse models (55). The MVA genome lacks functional copies of many genes that normally interfere with the host response to infection. Nevertheless, despite the strong inflammatory reaction, the only significantly modified upstream regulator associated with type 1 IFN gene was downregulated and only detected at 72 h p.i. in the blood. This might reflect the presence of interferon resistance genes, such as E3L, which is still functional in MVA (56).

The co-expression network highlighted interactions between the innate effectors by revealing hubs within the kinetics of the global immune response. Notably, it allowed identification of critical immune mediators, such as TNFα and MDSCs. TNFα was expected to be a key factor of the innate response as it is a central pro-inflammatory cytokine. This cytokine is indeed produced and released by granulocytes and macrophages and delivers signals leading to tissue necrosis. In certain contexts, TNFα could activate Langerhans cells (6), and induce T cell responses (57). The recruitment of CD14^+^ MDSCs was unexpected, since these cells are described to be mainly associated with immunosuppressive processes, such as tumor immune evasion and lymphocyte suppression through the production of reactive oxygen species (58, 59). Nevertheless, similar MDSCs were observed in the blood in rMVA-SIV vaccinated macaques and were found to exert T CD8^+^ suppression *in vitro* (60). In vaccine development studies, the induction of MDSCs was associated with the use of adjuvants (61, 62). Our results show that the involvement of MDSCs occurred as early as 24 h p.i., indicating that the resolution of the anti-MVA response may start very early. Further studies would be necessary to define whether the biological signatures identified in this model would be shared with the responses to other vaccines.

Our original approach combining *in vivo* imaging, histology, flow cytometry, multiplex cytokine analysis, and transcriptomic analysis using tools derived from systems biology, such as correlation networks, shows that the vaccine-induced immune response is a continuum of events which influence and synergize with each other. Out of note, we took advantage of these bioinformatic tools to statistically capture, in an efficient manner, this expression continuum with a limited number of animals. These synergies correspond to a cascade of reactions that depend on the original stimulus. We showed that the magnitude of the response of early effectors, such as local granulocytes and TNFα release, are directly associated with the first steps of the orientation of the adaptive response in the draining LNs. Furthermore, MDSCs should be considered to be a potential component of the signature of the response. Identification of such signatures should improve our understanding on how to effectively orientate the immune response, and could contribute to rational vaccine development.

## Ethics Statement

Adult male cynomolgus macaques (*Macaca fascicularis*), imported from Mauritius, weighing from 4 to 6 kg, were housed in the CEA animal facilities (Fontenay-aux-Roses, France). The macaques were handled in accordance with national regulations (Permit Number A 92-32-02), in compliance with the Standards of the Office for Laboratory Animal Welfare (OLAW) under Assurance number A5826-01, and the European Directive (2010/63, recommendation No. 9). This project received the government authorization Number 12-013. Interventions were performed by veterinarians and staff of the “Animal Science and Welfare” core facility after sedation with ketamin hydrochloride (10 mg/kg, Imalgen^®^, Rhône-Mérieux, Lyon, France).

## Author Contributions

Conceptualization: PR, YL, RG, FM, and CJ; methodology: PR, ND-B, A-SB, CC, RG, FM, and CJ; validation: PR, CC, RG, and FM; formal analysis: PR and NT; investigation: PR, LS, and HH; resources: NT and HH; writing—original draft: PR; writing—review and editing: PR, NT, LS, RG, FM, and CJ; funding acquisition: YL and RG; and supervision: YL, CC, RG, and FM.

## Conflict of Interest Statement

The authors declare that the research was conducted in the absence of any commercial or financial relationships that could be construed as a potential conflict of interest.
