# Supplementary material for: Molecular and Cellular Dynamics in the Skin, the Lymph Nodes, and the Blood of the Immune Response to Intradermal Injection of Modified Vaccinia Ankara Vaccine
Source: Front Immunol. 2018 Apr 25;9:870. doi: 10.3389/fimmu.2018.00870 (PMC5996922; doi:10.3389/fimmu.2018.00870)
Supplement: Supplementary file 1 [file presentation_1.PDF]

Supplementary Material

Molecular and Cellular Dynamics in the Skin, the Lymph Nodes and the Blood of the Immune Response to Intradermal Injection of Modified Vaccinia Ankara Vaccine

Pierre Rosenbaum, Nicolas Tchitchek, Candie Joly, Lev Stimmer, Hakim Hocini, Nathalie Dereuddre-Bosquet, Anne-Sophie Beignon, Catherine Chapon, Yves Levy, Roger Le Grand and Frédéric Martinon*

* Correspondence: Frédéric Martinon, frederic.martinon@cea.fr

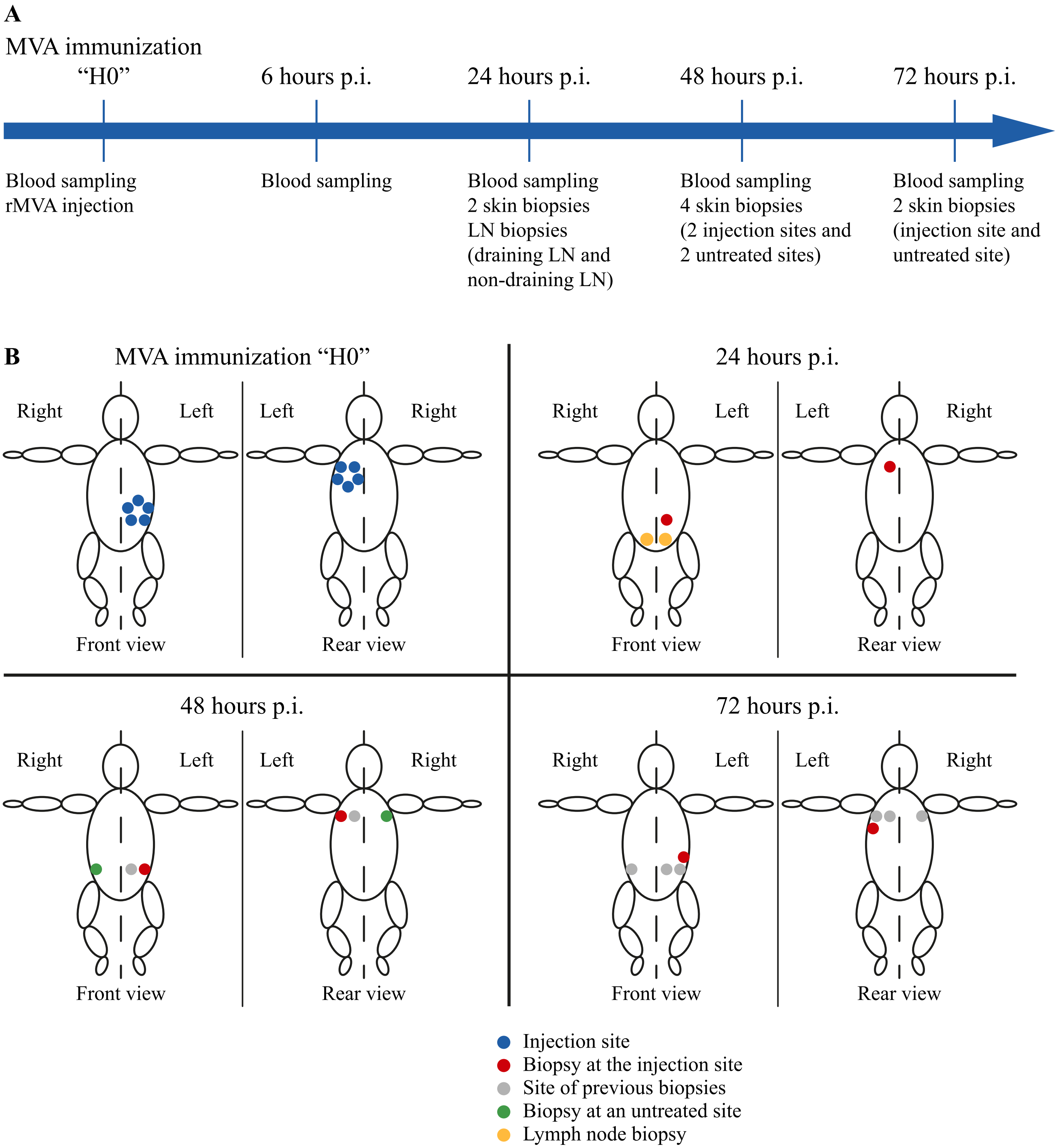

Supplementary Figure 1. Experimental design of tissue sampling procedure post MVA-GFP immunization. Non-human primates were immunized with ten intradermal injections of 4.10^7 PFU of MVA-GFP. (A) Timeline of blood and tissue sampling. (B) Localization of the immunization sites and tissue sampling.

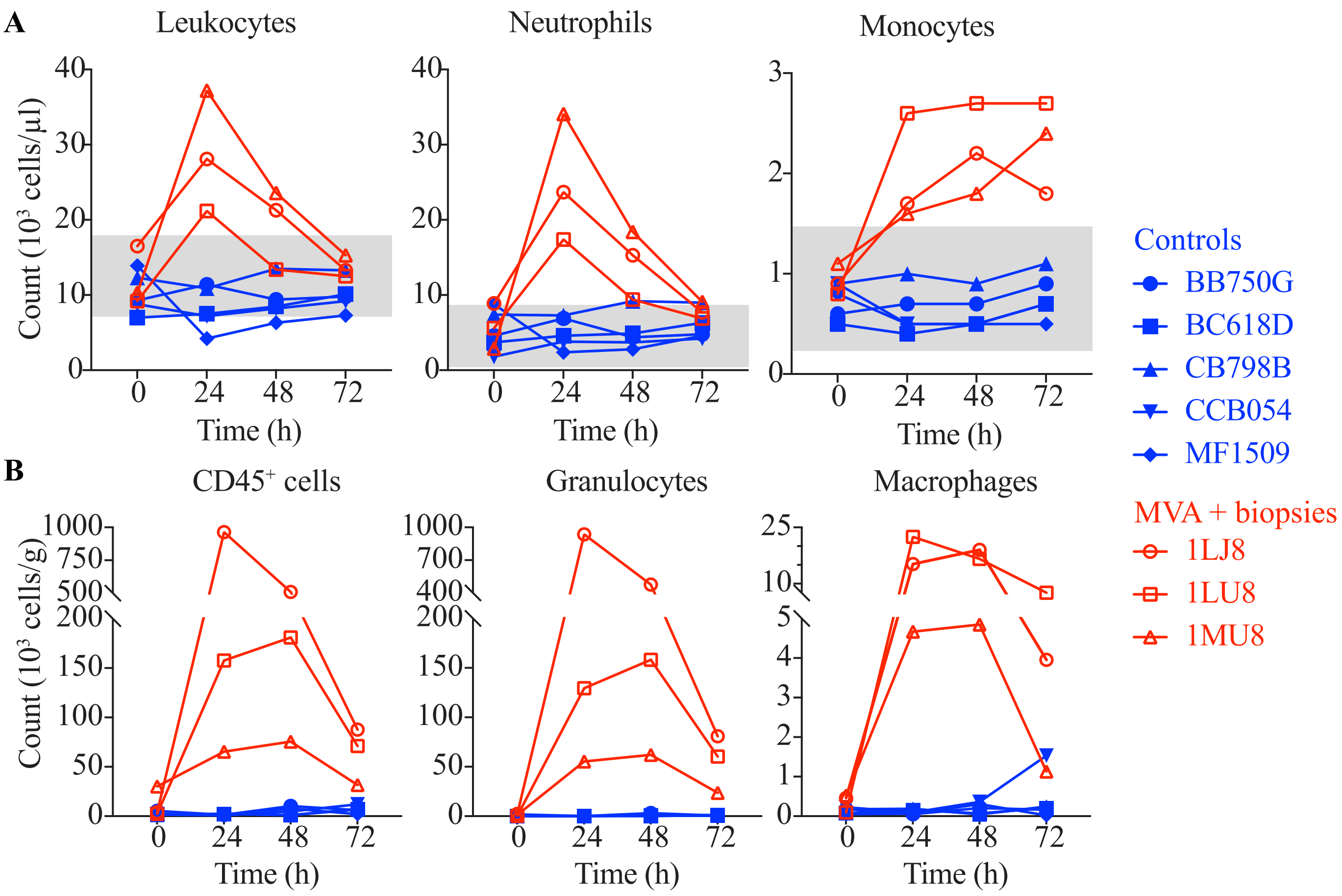

Supplementary Figure 2. Skin biopsies had insignificant effects on local and systemic inflammation in comparison with i.d. injection of MVA. (**A**) Complete blood count of control animals (blue) which experienced skin biopsies without i.d. injections and MVA-injected animals (red). In both groups of animals, the biopsies were performed as indicated in Sup. Figure 1. Grey areas correspond to normal values in Cynomolgus macaques (1). (**B**) Evaluation by flow cytometry of main inflammatory cell recruitment in the skin, for both groups of animals.

1. Xie L, Xu F, Liu S, Ji Y, Zhou Q, Wu Q, et al. Age- and sex-based hematological and biochemical parameters for Macaca fascicularis. PLoS One (2013) 8(6):e64892. Epub 2013/06/14. doi: 10.1371/journal.pone.0064892.

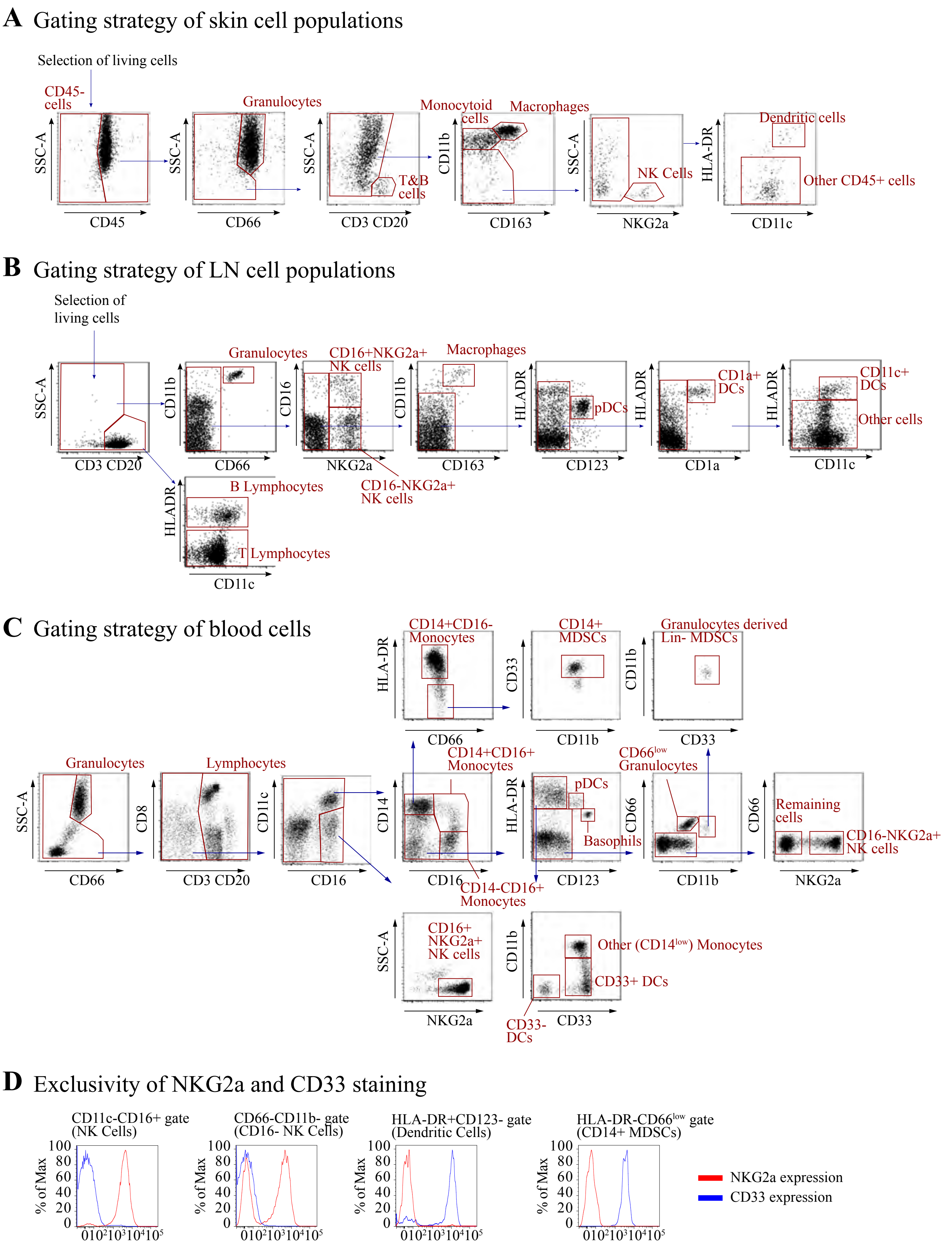

Supplementary Figure 3. Gating strategies for flow cytometry analysis. (**A**) Dot plots showing the gating strategy for skin cells. Eight-millimeter skin biopsies were cut and digested in collagenase to obtain a cell suspension. Cells were then stained using a 12-color panel. Doublets and debris were removed based

on FSH-H, FSC-A, and SSC-A. Living cells were selected based on differential live/dead blue cell staining. (**B**) Characterization of Immune-cell populations in lymph nodes using multiparameter flow cytometry. Single lymph nodes were cut and digested in collagenase to obtain a cell suspension. Cells were then stained using a 12-color panel. The indicated gating strategy was applied after doublet and debris removal based on FSH-H, FSC-A, and SSC-A. Living cells were selected based on differential live/dead blue cell staining. (**C**) Characterization of immune-cell populations in the blood using multiparameter flow cytometry. One hundred microliters of blood were stained using an 11-color panel. The indicated gating strategy was applied after doublet and debris removal based on FSH-H, FSC-A, and SSC-A. (**D**) Overlay representation of CD33 and NKG2a expression. We used one single channel (PE) to analyze both CD33 and NKG2a expression. We show that those markers are exclusive, and thus are not expressed by the same cell populations.

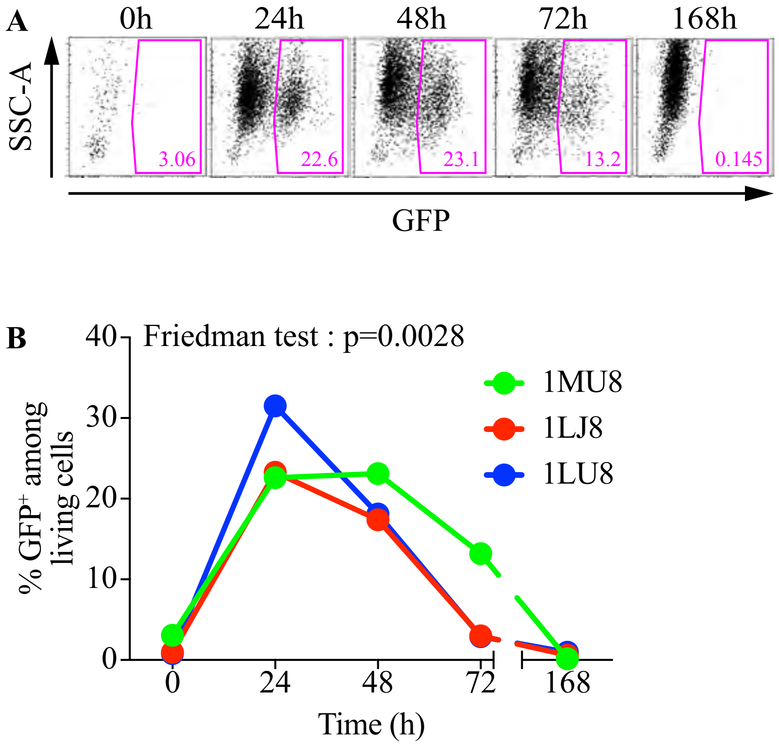

Supplementary Figure 4. Expression of GFP in skin cells. (**A**) Flow cytometry dot plots show the percentage of GFP+ cell over time among living cells from a digested skin biopsy (n = 3). One representative experiment of three is shown. (**B**) Evolution of the percentage of GFP+ cells among living cells in the three animals.
